# Impact of dexamethasone-sparing regimens on delayed nausea caused by moderately or highly emetogenic chemotherapy: a meta-analysis of randomised evidence

**DOI:** 10.1186/s12885-019-6454-y

**Published:** 2019-12-30

**Authors:** Luigi Celio, Erminio Bonizzoni, Emma Zattarin, Paolo Codega, Filippo de Braud, Matti Aapro

**Affiliations:** 1 Medical Oncology Unit 1, Fondazione IRCCS “Istituto Nazionale dei Tumori”, Via Venezian 1, 20133 Milan, Italy; 2 Section of Medical Statistics, Biometry and Epidemiology, University of Milan, Campus Cascina Rosa, Via Augusto Vanzetti 5, 20133 Milan, Italy; 3 Medical Affairs Department, Italfarmaco SpA, Via dei Lavoratori 54, 20092 Cinisello Balsamo, Italy; 4 Cancer Center, Clinique de Genolier, Route du Muids 3, 1272 Genolier, Switzerland

**Keywords:** Meta-analysis, Palonosetron, Dexamethasone, Moderately emetogenic chemotherapy, AC, Nausea, Emesis

## Abstract

**Background:**

Nausea can be particularly prominent during the delayed period. Therefore, we performed a meta-analysis of the available randomised evidence to assess the average effect of palonosetron plus one-day dexamethasone (DEX; also called the DEX-sparing strategy) compared with palonosetron plus 3-day DEX for control of chemotherapy-induced nausea and vomiting (CINV), focusing on delayed nausea.

**Methods:**

Eligible studies were identified through MEDLINE, Embase, and CENTRAL. Data on acute and delayed CINV were collected. Efficacy end points were complete response (CR; no vomiting, and no use of rescue medication), complete protection (CP; CR plus no clinically significant nausea), and total control (TC; CR plus no nausea) during the delayed period (days 2–5 after chemotherapy initiation). All randomised studies comparing palonosetron plus single-dose DEX (with or without another active agent) on day 1 followed by either no further DEX or additional DEX doses (both alone or in combination with another active agent) qualified.

**Results:**

Of 864 citations screened, 8 studies with 1970 patients were included in the meta-analysis. During the delayed period, the combined odds ratio (OR) for all comparisons was 0.92 (95% confidence interval [CI], 0.76–1.12) for CR, 0.85 (95% CI, 0.71–1.03) for CP, and 0.92 (95% CI, 0.77–1.11) for TC in patients undergoing moderately emetogenic chemotherapy (MEC) or anthracycline and cyclophosphamide-containing chemotherapy (AC). The absolute risk difference (RD) computations for all end points in the delayed period did not exceed the threshold of − 4% (range, − 1% to − 4%). The effect was similar in subgroups defined by various study design parameters. The absolute RD computations in the acute period did not exceed the threshold of 1% (range, 0 to 1%). For one-day vs. 3-day DEX, numbers needed to be treated in order for one additional patient to not experience CR, CP and TC over the delayed period were 100, 25 and 50, respectively.

**Conclusions:**

This meta-analysis demonstrates that DEX-sparing regimens do not cause any significant loss in protection against not only vomiting but also nausea induced by single-day MEC or AC during the delayed period. These data should lead clinicians to optimise use of prophylactic DEX in clinical practice.

## Background

Chemotherapy-induced nausea and vomiting (CINV) remain among the most common disturbing side effects of cancer chemotherapy [1, 2]. CINV is typically categorized as acute (within the first 24 h after chemotherapy initiation) and delayed (starts on day 2 and can have a time span of up to 6–7 days). In the last two decades, better prevention of CINV has been the result of several steps including the development of new classes of anti-emetics, such as 5-hydroxytryptamine type 3 receptor antagonists (5-HT_3_RAs) and neurokinin-1 receptor antagonists (NK-1RAs) [3]. Nevertheless, dexamethasone (DEX), one of the first antiemetic drug to be introduced, remains used extensively in multi-drug regimens recommended for the prevention of acute and delayed CINV caused by highly or moderately emetogenic chemotherapy (HEC and MEC, respectively) [4, 5]. Although prophylactic DEX has been generally considered safe, its administration may be associated with a wide range of side effects [6]. In a prospective survey patients receiving a multiple-day DEX regimen against delayed CINV caused by MEC reported several moderate-to-severe DEX-related side effects, including insomnia, abdominal symptoms, agitation, weight gain, skin rash and other symptoms in the week following chemotherapy [7]. Therefore, there is interest in minimising dose and frequency of the steroid, particularly in those patients who experience DEX-related side effects or in those with pre-existing conditions (like diabetes) that may be exacerbated by corticosteroids.

Palonosetron is a “second-generation” 5-HT_3_RA with a longer half-life (> 40 h) and distinct pharmacological properties compared with older agents in the 5-HT_3_RA class [8]. The unique pharmacology of palonosetron has been thought to partly explain its improved efficacy against delayed CINV [9]. Two randomised trials challenged the hypothesis that palonosetron plus single-dose DEX, also called the DEX-sparing strategy, is not inferior to palonosetron plus 3-day DEX against CINV caused by MEC [10, 11]. These studies met their goal, but the study by Aapro et al. [10] included only breast cancer patients receiving the combination of an anthracycline and cyclophosphamide (AC), whereas the study by Celio et al. [11] included a wide range of MEC regimens including AC. At the time the studies were performed AC was considered as MEC, and only successively classified as HEC, for which the addition of an NK-1RA is recommended for CINV control [4, 5]. The international guidelines have recently changed the anti-emetic management of AC-type chemotherapy in female patients and of MEC, and no longer recommend DEX for the prevention of delayed CINV, except for agents with known potential for delayed symptoms [4, 5]. Interestingly, this recommendation stems from the lack of convincing evidence to support the benefit of DEX against delayed CINV caused by AC or MEC, before a formal meta-analysis. An important consideration when evaluating data from the first DEX-sparing trials is that, although any potentially detrimental effect on antiemetic protection would be expected to occur during the delayed period, the studies used data from the overall observation period (i.e., acute plus delayed periods) to calculate the primary efficacy outcome of complete response (CR) [10, 11]. It is also important to highlight that CR is a composite end point that does not include any direct assessment of nausea whose optimal control still remains a treatment challenge [12]. Therefore, there were several important questions unanswered regarding the exact therapeutic impact of the DEX-sparing strategy on the management of CINV. Specifically, how effective is the DEX-sparing strategy compared with a multiple-day DEX regimen against delayed CINV? Is the effect different when control of nausea is assessed? What evidence exists for the efficacy of DEX-sparing strategy when combined with other active agents? Is there evidence of variability in the reported protective effect in patients receiving HEC or MEC? Finally, is there evidence of any distinct tolerability profile of the DEX-sparing strategy? In order to help answer these questions, we report the results of a systematic review and meta-analysis of randomised controlled trials (RCTs) addressing the efficacy of DEX-sparing strategy for the prevention of delayed CINV, and specifically nausea.

## Methods

### Literature search

A literature search to identify RCTs assessing the DEX-sparing strategy in CINV was initially conducted in the MEDLINE database via Pubmed. In a second step the search was extended to include EMBASE and CENTRAL (Cochrane Central Register of Controlled Trials) using criteria described below. Since the first RCT assessing the DEX-sparing strategy against CINV was fully reported in 2010 [10], our search covered the time period from January 1, 2010 to June 30, 2018. The DEX-sparing strategy relies on the use of palonosetron and therefore free text keyword “palonosetron” was used to prompt relevant literature; the search was also limited to English language clinical trials. For manual search, we examined the reference lists of included RCTs and past meta-analyses. The search results were combined to yield a common set of citations from which the titles and abstracts were screened for potential qualifying studies, and then full-text review identified studies that were qualified for this review based on pre-specified inclusion criteria presented below.

### Selection criteria

We limited our selection to titles and abstracts if the study: 1) was a randomised trial of adult subjects, 2) compared palonosetron plus single-dose DEX (with or without another active agent) to the same anti-emetic regimen on day 1 followed by either no further DEX or additional DEX doses (both alone or in combination with another active agent) on the subsequent days for the prevention of CINV following single-day chemotherapy regimens, and 3) reported relevant clinical outcome data. Studies were included if at least one efficacy end point [i.e., complete response (CR), complete protection (CP), or total control (TC)] in the acute or delayed periods was available. Because of the high likelihood of carry-over effects on anti-emetic efficacy over the delayed period [13], we decided a priori to include only studies in which the same anti-emetic regimen for prevention of acute CINV was administered to both investigational and control arms. If a study included multiple cycles of chemotherapy, only the results from cycle 1 were considered. Cross-over studies qualified only if the first-cycle data were reported and usable. Studies were also excluded if duplicates of articles already part of the database, or if consisted in case reports or clinical observations.

### Definition of outcomes

The primary objective was the prevention of CINV during the delayed period (i.e., day 2 through 5 after chemotherapy initiation) with special attention to nausea data. It was evaluated through the proportion of patients who achieve CR (no vomiting, and no use of rescue medication), CP (CR plus no significant nausea [< 25 mm on a visual analogue scale (VAS) or no more than mild nausea on a visual categorical scale]), and TC (CR plus no nausea [VAS < 5 mm]). Secondary objectives were control of CINV during the acute study period (within 24 h after chemotherapy) and the proportion of patients free from side effects associated with prophylactic DEX [7].

### Data extraction and quality assessment

Two investigators (LC and E Z) independently conducted literature search and extracted data from studies that met the pre-specified inclusion criteria for meta-analysis. For multiple articles concerning a given study, only the study presenting the most complete data was included. Discrepancies were handled through discussion and consensus. For two qualified studies, the rates of acute and delayed CP and/or TC were not reported in the full-text articles [10, 11], but were calculated by accessing the database of each study. We assessed the emetogenic risk of one specific agent in included studies according to the latest emetic-risk classification of antineoplastic agents [4, 5]. In light of this, for the study by Celio et al. [11] that included patients receiving either MEC or AC, only data from the MEC group were analysed in this meta-analysis. Since currently there are no data on the efficacy of DEX-sparing strategies in the cisplatin setting, for the study by Ito et al. [14] that included patients receiving either AC or cisplatin, only data from the AC group were analysed. The study by Roila et al. [13] evaluated the efficacy of palonosetron and single-dose DEX plus 3-day aprepitant versus palonosetron and single-dose aprepitant (125 mg on day 1) plus 3-day DEX in patients who were receiving AC for breast cancer. We decided to include this study in the meta-analysis based on the results of the study by Herrington et al. [15]. This double-blind, pilot study demonstrated that a single dose of aprepitant 125 mg has similar efficacy as the 3-day aprepitant regimen, when both are administered in combination with palonosetron and multiple DEX doses, in patients who are receiving HEC regimens. In addition to the main end points, the following information was searched in each study: definition of the condition of interest and of the outcome of interest, type of chemotherapy (HEC or MEC), cancer types, patient demographics such as the average age and sex ratio in the study, comparator (DEX given either alone or in combination with other active agent in the delayed period), concurrent background anti-emetics given to all patients, and sponsorship/funding for trial as stated in the publication. Since no significant difference in efficacy between the doses of palonosetron 0.25 mg and 0.75 mg has been reported [16], no distinction was made regarding the amount of palonosetron dosage. If the tolerability data were reported separately for each specific day of the observation period, we agreed a priori to select the incidence rates of the worst day over the delayed period for inclusion to the meta-analysis calculations.

We assessed the risk of bias of each study using the Cochrane Collaboration’s tool [17]. Each eligible study was assessed on the basis of selection bias (method of random allocation and adequacy of concealment), performance bias (blinding of the investigators and patients to the investigational treatment), detection bias (blinding of outcome assessment), attrition bias (incomplete outcome data), reporting bias (selective outcome reporting), and other sources of bias. The studies were considered high risk if they exhibited possibility of high risk of bias in at least one of the criteria.

### Data synthesis

Data synthesis was performed using the software package Review Manager version 5.3 (the Cochrane Collaboration). Results are reported in accordance with Preferred Reporting Items for Systematic Reviews and Meta-analyses (PRISMA) guidelines [18] (the protocol was not registered) and expressed as odds ratio (OR) effect measures, absolute risk differences (RDs), and accompanying 95% confidence intervals (CIs). The Mantel-Haenszel method was applied and random-effects models were used that provide a more conservative estimate of effect. For comparison, fixed-effect models were also performed but did not lead to any diverging conclusions (data not shown). An intention-to-treat analysis was applied within each clinical trial. The inverse of the RD provides the metric number needed to harm (NNH), which may be useful for the clinical interpretation of the results [19]. The proportion of variation due to heterogeneity rather than due to chance was tested by the Q statistic and the I^2^ statistic. Significant heterogeneity was considered to be present if the *P*-value was < 0.10 and I^2^ was 50% or more [20]. A *P*-value of less than 0.05 was considered statistically significant in the test for overall effect. Since less than 10 studies were included in the meta-analysis, we did not examine the presence of small-study effects graphically by generating funnel plots. Patient approval was waived because of the nature of the study.

We assessed the robustness of the results for primary objective by various types of subgroup analyses. The effect of the methodological quality of the studies was assessed by subgroups: 1) comparing unblinded studies with single-blind and double-blind studies, and small studies (≤100 patients per arm) with large studies. Subgroup analyses were also performed to explore possible relationships between known covariates (emetogenicity of chemotherapy, female sex, and use of NK-1RA) and treatment effects. Emetogenicity subgroup analyses were based on the fact that the combination of AC is now classified as HEC [4, 5].

For the meta-analysis of tolerability data, we mainly took into account the study by Ito et al. [14] that lists a number of DEX-related side effects. This study allowed us to identify side effects reasonably caused by the corticosteroid in the included studies. A quantitative synthesis of DEX-related side effects reported in qualified studies was to be conducted if the proportion of patients not experiencing a specific side effect was reported in at least three studies. For the purpose of meta-analysis, the actual proportion of patients free from side effects was obtained by subtracting the proportion of patients experiencing the side effect from one.

## Results

**Fig. 1:**
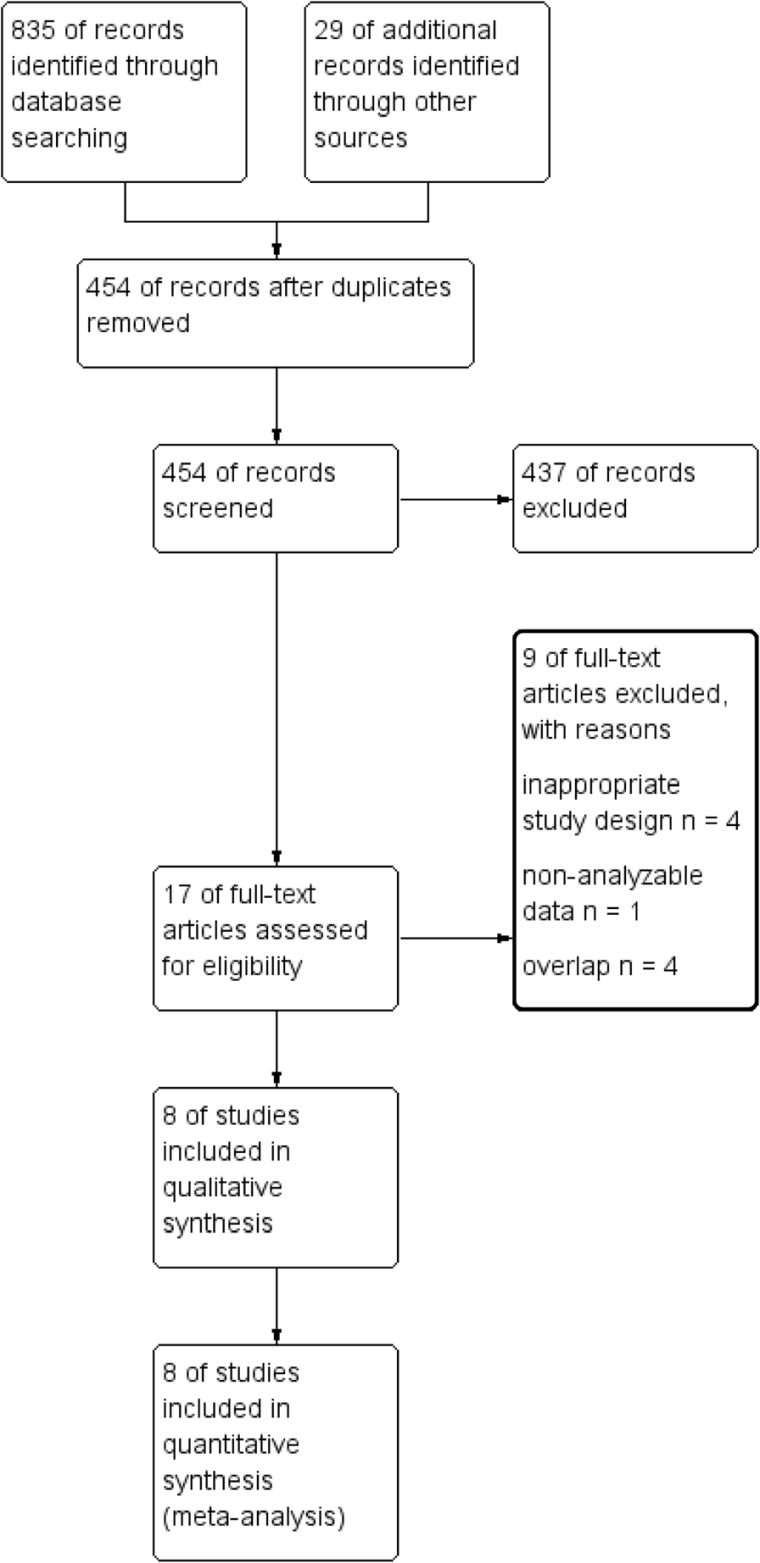
PRISMA flow chart of search strategy and study selection

Electronic and manual literature searches identified 17 potentially useful RCTs (Fig. 1); among these, 8 studies were not eligible for the meta-analysis (details are shown in Additional file 1: Table S1). From the 9 retrieved studies, 1 was excluded from the meta-analysis [21]: a four-arm RCT including anti-emetic regimens consisting of palonosetron plus DEX on day 1 of chemotherapy, followed by prochlorperazine with or without DEX on days 2 and 3, because of non-analyzable data. This study may be less important because prochlorperazine is not anymore guideline-recommended agent for the prevention of CINV.

### Characteristics of included randomised controlled studies

A total of 8 studies with 8 pertinent comparisons were included in the meta-analysis (Table 1) [10, 11, 13, 14, 22–25]. The studies have been published from 2010 to 2018. Withdrawals, drop-outs and losses to follow up were stated in all studies and accounted for less than 7% of each study population. In the meta-analysis, the total number of assessable patients was 1970. The majority of the included studies were multicenter (75%); they were conducted in Europe (38%) or Japan (62%). All but 3 studies [10, 13, 25] included patients with various types of solid cancer; none of the included studies reported the mean (median) age of their population to be less than 50 years (Table 1). Five studies recruited exclusively female patients [10, 13, 22, 23, 25], while all studies included patients who had not been exposed to the chemotherapy before. In 4 of the 8 comparisons no-treatment controls vs. DEX during the delayed period were evaluated [11, 22–24], while in 2, an NK-1RA was prescribed to both treatment arms [14, 25]. Four pertinent studies involved HEC [10, 13, 14, 25], essentially the combination of AC (93% of patients), whereas there were 3 comparisons [22–24] in which only MEC regimens were considered (Table 1). In the MEC studies, the majority of patients (82%) received carboplatin- or oxaliplatin-based chemotherapy [11, 22–24].

**Table 1:** Characteristics of randomized controlled studies included in the meta-analysis

Author/year [reference]	Study design	Intervention (dose in mg)	No. of patients^a^	Type of cancer^b^	Type of chemotherapy	Female (%)	Mean age (years)	Alcohol non-users (%)	Chemo-naive (%)
Aapro/2010 [10]	Multicenter, double-blind, non-inferiority, parallel	1) Palo (0.25) + DEX (8) on day 1	1) 151/151	Breast	AC	1) 100	1) 52.1	1) 79.8	1) 100
		2) Palo (0.25) + DEX (8) on day 1 + DEX (8) on days 2–3	2) 149/149			2) 100	2) 51.2	2) 80.2	2) 100
Celio/2011 [11]	Multicenter, open-label, non-inferiority, parallel	1) Palo (0.25) + DEX (8) on day 1	1) 166/163	Breast, colon, lung	MEC (*n* = 237)^c^ or AC	1) 62.0	1) 56.9	1) 60.8	1) 100
		2) Palo (0.25) + DEX (8) on day 1 + DEX (8) on days 2–3	2) 166/161			2) 68.1	2) 57.2	2) 59.6	2) 100
Roila/2014 [13]	Multicenter, double-blind, superiority, parallel	`1) Palo (0.25) + APR (125) + DEX (8) on day 1 + APR (80) on days 2–3	1) 289/278	Breast	AC	1) 100	1) 53.1	1) 83.5	1) 100
		2) Palo (0.25) + APR (125) + DEX (8) on day 1 + DEX (8) on days 2–3	2) 291/273			2) 99.3	2) 52.9	2) 76.9	2) 100
Furukawa/2015 [22]	Single-center, open-label, non-inferiority, parallel	1) Palo (0.75) + DEX (20) on day 1	1) 44/43	Ovary, endometrium, cervix	MEC (carboplatin)	1) 100	1) 59^d^	1) 90.7	1) 100
		2) Palo (0.75) + DEX (20) on day 1 + DEX (8) on days 2–3	2) 44/39			2) 100	2) 62^d^	2) 92.3	2) 100
Matsuura/2015 [23]	Multicenter, open-label, superiority, parallel	1) Palo (0.75) + DEX (9.9 or 20) on day 1	1) 58/56	Ovary, endometrium, cervix	MEC (carboplatin)	1) 100	1) 57.7	1) 69.6	1) 100
		2) Palo (0.75) + DEX (9.9 or 20) on day 1 + DEX (8) on days 2–3	2) 58/53			2) 100	2) 56.7	2) 64.2	2) 100
Komatsu/2015 [24]	Multicenter, open-label, non-inferiority, parallel	1) Palo (0.75) + DEX (9.9) on day 1	1) 154/151	NR	MEC (mainly oxaliplatin or irinotecan)	1) 43.0	1) 64.1	1) 51.0	1) 100
		2) Palo (0.75) + DEX (9.9) on day 1 + DEX (8) on days 2–3	2) 154/154			2) 43.5	2) 64.0	2) 51.9	2) 100
Kosaka/2016 [25]	Single-center, single-blind, superiority, parallel	1) Palo (0.75) + DEX (12) + APR (125) on day 1 + APR (80) on days 2–3	1) 41/39	Breast	AC	1) 100	1) 52.6	1) 64.1	1) 100
		2) Palo (0.75) + DEX (12) + APR (125) on day 1 + APR (80) + DEX (8) on days 2–3	2) 41/41			2) 100	2) 53.5	2) 61.0	2) 100
Ito/2018 [14]	Multicenter, double-blind, non-inferiority, parallel	1) Palo (0.75) + DEX (9.9) + NK-1RA^f^ on day 1 + APR (80) on days 2–3	1) 200/200	Breast, oesophagus, stomach	AC (*n* = 306)^e^ or cisplatin	1) 81.5	1) 54.1^d^	1) NR	1) 100
		2) Palo (0.75) + DEX (9.9) + NK-1RA on day 1 + APR (80) + DEX (8) on days 2–3	2) 201/196			2) 80.1	2) 55^d^	2) NR	2) 100

Abbreviations: *Palo* palonosetron, *DEX* dexamethasone, *APR* aprepitant, *AC* anthracycline plus cyclophosphamide, *MEC* moderately emetogenic chemotherapy, *NK-1RA* neurokinin-1 receptor antagonist, *NR* not reported

^a^patients randomised/patients included in efficacy analyses

^b^main types of malignancies

^c^only patients receiving chemotherapy regimens classified as MEC were analysed in the meta-analysis

^d^median age

^e^only patients receiving AC-based regimens were analysed in the meta-analysis

^f^patients also received single-dose fosaprepitant on day 1 rather than aprepitant for 3 days

We classified 4 unblinded studies [11, 22–24] at a high risk of detection bias (as shown in Additional file 2: Figure. S1). Regarding potential funding bias, we found that 1 study had been funded by industry [10], 1 was unfunded [11], 1 had mixed funding [13], and 1 study had been funded by other group not industry [24]. The source of funding was not stated for 4 studies [14, 22, 23, 25], but only for one of them authors declared conflicts of interest [14].

### Efficacy in the delayed period

**Fig. 2:**
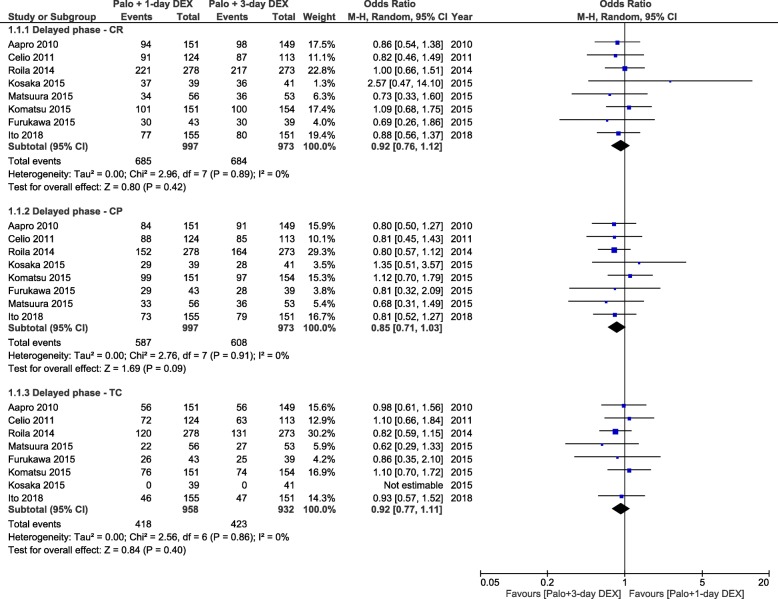
Forest plot of combined odds ratios for anti-emetic efficacy of 1-day versus 3-day dexamethasone during the delayed period. Abbreviations: CR, complete response, CP, complete protection, TC, total control, Palo, palonosetron, DEX, dexamethasone

Results for CR and CP during the delayed period were available for 8 studies (1970 patients), while those for TC were available only for 7 studies (1890 patients) [10, 11, 13, 14, 22–24]. The combined ORs demonstrated no statistically significant difference between the anti-emetic regimens for all end points: CR (OR = 0.92, 95% CI, 0.76 to 1.12), CP (OR = 0.85, 95% CI, 0.71 to 1.03) and TC (OR = 0.92, 95% CI, 0.77 to 1.11; Fig. 2).The absolute RD computations for all three end points in the delayed period did not exceed the threshold of − 4% (range, − 1 to − 4%) (Table 2). The NNHs indicated that about one in 100 patients treated with DEX-sparing regimens will not experience a CR, about one in 25 patients will not experience a CP and about one in 50 patients will not have a TC during the delayed period.

**Table 2:** Absolute risk differences between 1-day and 3-day DEX intervention arms for efficacy end points

End point	Absolute RD (%)	95% CI	*P* for overall effect	*P* for heterogeneity
All studies				
CR, acute period	0	−2 to 3	0.87	0.61
CR, delayed period	−1	−5 to 3	0.64	0.82
CP, acute period	0	−3 to 3	0.84	0.56
CP, delayed period	−4	−8 to 1	0.10	0.89
TC, acute period	1	−3 to 4	0.72	0.64
TC, delayed period	−2	−6 to 3	0.40	0.86
MEC studies^a^				
CR, acute period	1	−2 to 5	0.38	0.56
CR, delayed period	−2	−9 to 4	0.48	0.75
CP, acute period	1	−4 to 5	0.74	0.25
CP, delayed period	−2	−9 to 4	0.51	0.69
TC, acute period	0	−5 to 6	0.90	0.31
TC, delayed period	0	−8 to 7	0.89	0.59
AC studies				
CR, acute period	−2	−6 to 2	0.36	0.50
CR, delayed period	0	−5 to 5	0.94	0.53
CP, acute period	0	−6 to 5	0.87	0.52
CP, delayed period	−4	−10 to 1	0.11	0.76
TC, acute period	1	−5 to 7	0.69	0.73
TC, delayed period	−3	−8 to 3	0.34	0.79
Studies without an NK1-RA				
CR, acute period	1	−2 to 5	0.37	0.77
CR, delayed period	−3	−8 to 3	0.35	0.87
CP, acute period	1	−3 to 5	0.59	0.33
CP, delayed period	−3	−9 to 3	0.29	0.79
TC, acute period	0	−5 to 5	0.99	0.45
TC, delayed period	0	−6 to 6	0.87	0.75
Studies with an NK-1RA^b^				
CR, acute period	−3	−8 to 2	0.27	0.38
CR, delayed period	1	−5 to 6	0.83	0.42
CP, acute period	−2	−8 to 4	0.55	0.48
CP, delayed period	−4	−10 to 2	0.19	0.56
TC, acute period	2	−4 to 9	0.51	0.61
TC, delayed period	−3	−10 to 3	0.29	0.62
Mixed studies				
CR, acute period	3	−2 to 8	0.23	0.32
CR, delayed period	−1	−8 to 7	0.85	0.47
CP, acute period	3	−2 to 9	0.26	0.24
CP, delayed period	−1	−8 to 7	0.86	0.39
TC, acute period	2	−7 to 11	0.64	0.19
TC, delayed period	2	−6 to 11	0.59	1.00
Only-women studies				
CR, acute period	−1	−4 to 2	0.55	0.75
CR, delayed period	−1	−5 to 3	0.66	0.67
CP, acute period	−1	−5 to 2	0.48	0.75
CP, delayed period	−5	−10 to 0	0.06	0.92
TC, acute period	0	−4 to 5	0.91	0.65
TC, delayed period	−1	−6 to 4	0.62	0.39

Abbreviations: *DEX* dexamethasone, *RD* risk difference, *CI* confidence interval, *CR* complete response, *CP* complete protection, *TC* total control, *MEC* moderately emetogenic chemotherapy, *AC* anthracycline and cyclophosphamide, *NK-1RA* neurokinin-1 receptor antagonist

^a^all patients received chemotherapy regimens classified as MEC

^b^all patients received the combination of AC

A RD below 0 (negative absolute difference) favours the 3-day DEX intervention arm and a RD above 0 (positive absolute difference) favours the 1-day DEX intervention arm

### Efficacy in the acute period

**Fig. 3:**
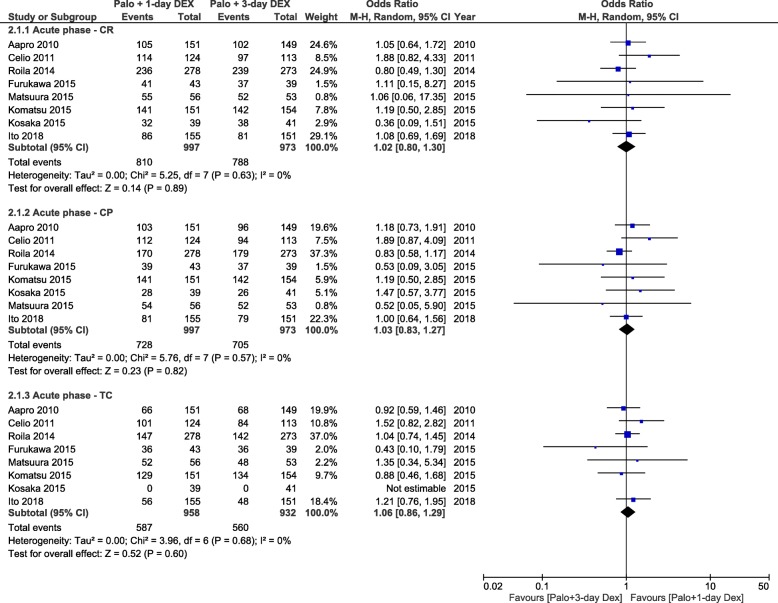
Forest plot of combined odds ratios for anti-emetic efficacy of 1-day versus 3-day dexamethasone during the acute period. Abbreviations: CR, complete response, CP, complete protection, TC, total control, Palo, palonosetron, DEX, dexamethasone

Results for CR and CP during the acute period were available for eight studies (1970 patients), while those for TC were available only for 7 studies (1890 patients). The combined ORs demonstrated no statistically significant difference between the anti-emetic regimens for all three end points (Fig. 3). The absolute RD computations for all three end points in the acute period did not exceed the threshold of 1% (range, 0 to 1%) (Table 2).

### Subgroup analyses

Subgroup analyses showed consistent patterns across subgroups in the delayed period. In random-effects models, no significant interaction existed between the subgroups of MEC and AC (test for subgroup differences: Chi^2^ = 0.04, *P* = 0.84; I^2^ = 0%; Chi^2^ = 0.23, *P* = 0.63; I^2^ = 0%; and Chi^2^ = 0.28, *P* = 0.60; I^2^ = 0%, respectively, for CR, CP, and TC; Additional file 3: Figures S2a, b, and c), unblinded and blinded studies (Chi^2^ = 0.04, *P* = 0.84; I^2^ = 0%; Chi^2^ = 0.23, *P* = 0.63; I^2^ = 0%; and Chi^2^ = 0.28, *P* = 0.60; I^2^ = 0%; Additional file 3: Figures S3a, b, and c), small and large studies (Chi^2^ = 0.15, *P* = 0.70; I^2^ = 0%; Chi^2^ = 0.00, *P* = 0.96; I^2^ = 0%; and Chi^2^ = 0.87, *P* = 0.35; I^2^ = 0%; Additional file 3: Figures S4a, b, and c), mixed and only-women studies (Chi^2^ = 0.13, *P* = 0.72; I^2^ = 0%; Chi^2^ = 0.76, *P* = 0.38; I^2^ = 0%; and Chi^2^ = 0.66, *P* = 0.42; I^2^ = 0%; Additional file 3: Figures S5a, b, and c), and studies with or without NK-1RA (Chi^2^ = 0.20, *P* = 0.66; I^2^ = 0%; Chi^2^ = 0.05, *P* = 0.82; I^2^ = 0%; and Chi^2^ = 0.51, *P* = 0.47; I^2^ = 0%; Additional file 3: Figures S6a, b, and c) in the delayed period.

### Tolerability

**Fig. 4:**
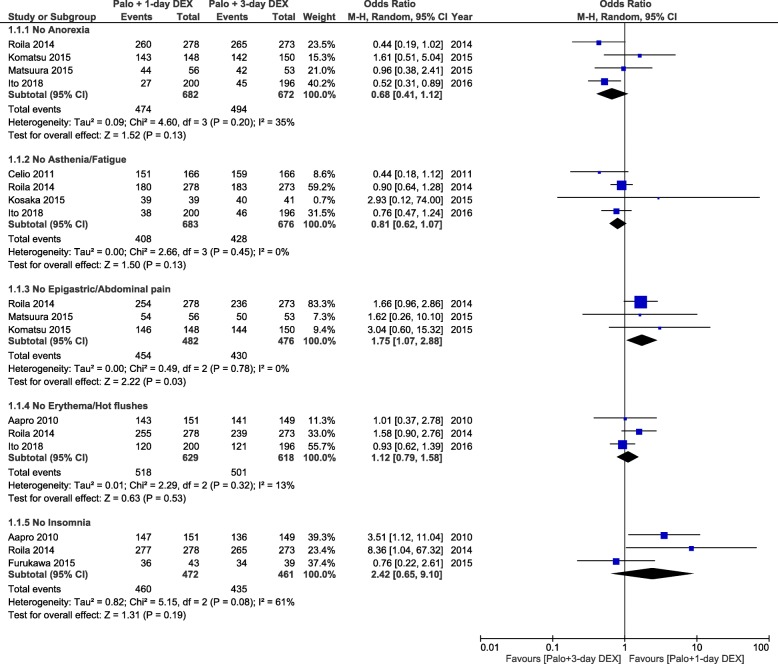
Forest plot of combined odds ratios for dexamethasone-related side effects in patients receiving 1-day or 3-day dexamethasone. Abbreviations: Palo, palonosetron, DEX, dexamethasone

For tolerability, the total number of assessable patients was 2148. All included studies provided some reporting of DEX-related side effects, but the quality of the reporting varied greatly. One study reported on DEX-related side effects that were pre-specified in the study protocol [14], while only two studies reported on side effects that occurred over the delayed period [13, 14]. Four studies reported on anorexia [13, 14, 23, 24], one on asthenia [13], and three studies reported on fatigue [11, 14, 25]. In the absence of significant heterogeneity, a multiple-day DEX regimen was not significantly better tolerated than the DEX-sparing strategy in terms of anorexia (OR = 0.68, 95% CI, 0.41 to 1.12) and asthenia/fatigue (OR = 0.81, 95% CI, 0.62 to 1.07; Fig. 4). However, only the study by Ito et al. [14] that included patients receiving AC- or cisplatin-based HEC showed that fatigue was significantly more frequent over the delayed period in single-dose DEX arm. One study reported on epigastric pain [13], and two studies on abdominal pain [23, 24]; one study reported on hot flushes [14], and two on erythema [10, 13]. Lastly, three studies reported on insomnia [10, 13, 22]. The DEX-sparing regimen was more favourable with respect to the occurrence of epigastric/abdominal pain (OR = 1.75, 95% CI, 1.07 to 2.88; Fig. 4). For insomnia, no significant difference was apparent between treatments (OR = 2.42, 95% CI, 0.65 to 9.10), but there was significant heterogeneity for the OR across studies. The heterogeneity was driven by the smallest study that showed no difference between the two DEX regimens. Therefore, the DEX-sparing strategy resulted in significantly lower frequency of insomnia than multiple DEX doses when the study by Furukawa et al. [22] was excluded (OR = 4.29, 95% CI, 1.57 to 11.6).

## Discussion

In the present meta-analysis, the total overall effect calculated for all included studies, regardless of whether patients were treated with MEC or AC HEC, shows that the DEX-sparing strategy does not cause any significant detrimental effect over a multiple-day DEX regimen for the protection against delayed CINV. The absolute RD computations for all three end points in the delayed period did not exceed the threshold of − 4%, with the lower boundary of the 95% CIs not exceeding − 8%. The results of cumulative meta-analyses indicate that 100 patients need to be treated with the DEX-sparing strategy to prevent one additional patient from experiencing CR over the delayed period. However, no use of rescue medication evaluated as part of the composite end point CR serves only as a surrogate marker for no nausea or only mild nausea [26]. Accordingly, reporting the control of nausea using other end points such as CP and TC that include also a direct assessment of nausea is of great clinical relevance, as nausea can be particularly prominent during the delayed period [26]. In cumulative meta-analyses, we found that 25 and 50 patients need to be treated to prevent one additional patient from experiencing CP and TC, respectively. In the subgroup of MEC studies, 50 patients need to be treated to cause no delayed CP in one additional patient, while there is virtually no RD between the two DEX regimens in terms of TC. In the AC HEC subgroup, 25 and 33 patients need to be treated to cause one additional patient not experiencing CP and TC, respectively. These results are very reassuring on the anti-emetic efficacy of DEX-sparing regimens, having been obtained in a heterogeneous population including breast cancer patients, a particularly high-risk subgroup for nausea caused by AC [26]. Overall, the results of the current meta-analysis constitute new and clinically relevant information that adds to the findings from a recent meta-analysis of individual patient data (IPD) showing that the DEX-sparing strategy is not associated with a significant loss in anti-emetic control during the overall period, irrespective of known risk factors for CINV [27]. It also should be noted that the IPD meta-analysis included patients from five studies of the DEX-sparing strategy, while there was only the small-size study by Kosaka et al. [25] which included patients undergoing AC who received also an NK-1RA as recommended by current guidelines [4, 5]. Furthermore, only data regarding the occurrence of no significant nausea (i.e., patients experiencing no more than mild nausea) were analysed in the IPD meta-analysis, while there were no data regarding the total control of nausea which allow to fully assess the impact of the DEX-sparing strategy on nausea control.

In the present meta-analysis, no evidence of variability in the treatment effect emerged. Several subgroup analyses also failed to identify signs of heterogeneity that might affect the reported effect in studies; i.e., patients treated with AC HEC or MEC, patients that received an NK-1RA or not, or patients that were men and women or only women. In light of this, it can be concluded that these subgroups did not estimate different population parameters. We also performed subgroup analyses to deal with concerns that the methodological quality of studies might affect the reported effect. These subgroup analyses failed to identify any significant interaction between the subtotal estimates for the subgroups of unblinded and blinded studies, and small- and large-sized studies for all end points. Because of the subjective nature of nausea, it is of special importance that the study design is blind. Therefore, the finding of no significant interaction between unblinded and blinded studies supports the robustness of cumulative results showing that there are no major concerns related to the protective effect of DEX-sparing strategy against delayed CINV. It is also important to underline that the experimental design of DEX-sparing studies involving the same prophylaxis against acute CINV in both treatments arms offers a unique opportunity to exclude that anti-emetic efficacy during the delayed period is related to carry-over effect from better control of acute CINV [13]. In the absence of significant heterogeneity, cumulative meta-analyses show no significant differences between the treatment arms for all efficacy end points during the acute period.

Some key findings of this meta-analysis deserve specific comments. Firstly, the evidence from the AC HEC subgroup indicates that the combination of palonosetron, an NK-1RA, and multiple DEX doses does not result in better protection against delayed CINV compared with the same regimen containing single-dose DEX. It is important to underline that the currently recommended three-drug prophylaxis in patients undergoing AC does not involve DEX against delayed CINV because pivotal trials of NK-1RAs did not use additional DEX doses during the delayed period [4, 5]. Secondly, the cumulative results in the MEC subgroup were obtained in studies in which the majority of patients received either carboplatin- or oxaliplatin-based regimens, two MEC settings that are at risk for delayed CINV. In patients undergoing carboplatin the guidelines now recommend the same three-drug prophylaxis as for AC HEC, while they recommend DEX against delayed CINV caused by MEC only for agents with known potential for delayed symptoms such as oxaliplatin [4, 5]. However, in the guidelines from the Multinational Association of Supportive Care in Cancer/European Society for Medical Oncology, this recommendation derives from the lack of evidence for the use of DEX on days 2 and 3 routinely during the delayed period [4]. Finally, we note that the DEX-sparing strategy has a greater impact on the achievement of CP rather than TC. This finding suggests that the main effect of a multiple-day DEX regimen against delayed nausea may increase the number of patients reporting mild nausea rather than patients free from nausea. A recent phase III study of patients receiving AC- or cisplatin-based HEC demonstrated that adding olanzapine to a triplet containing aprepitant/fosaprepitant, a 5-HT_3_RA, and DEX significantly increases the rate of no nausea during the delayed period [12]. DEX was administered for four consecutive days in each treatment arm of the study, but the results of the current meta-analysis question the clinical relevance of additional DEX doses for improving the control of delayed nausea in patients treated with AC and receiving a four-drug antiemetic regimen containing palonosetron, single-dose DEX, an NK-1RA, and olanzapine. In addition, in the olanzapine arm the proportion of patients free from acute CINV was significantly higher than that in the control arm, and this does not exclude the possibility that a carry-over effect can have partly affected the magnitude of the protective effect against delayed symptoms [12]. Interestingly, the protective effect of palonosetron, single-dose DEX, and olanzapine has been reported to be comparable to that of palonosetron, aprepitant, and multiple DEX doses against delayed CINV, except for nausea (there was a significant increase in nausea control with olanzapine), in patients receiving AC- or cisplatin-based HEC [28].

Short-term use of corticosteroids was recently reported to be associated with an unexpected increased risk of corticosteroid-related side effects in a population-based cohort study [29]. In the CINV setting, short-term DEX is administered repeatedly in cancer patients receiving multiple cycles of chemotherapy, which can substantially increase the risk of DEX-related side effects [7, 30–32]. In two recent prospective studies, prophylactic DEX had a negative impact on adrenal function or bone health in cancer patients undergoing consecutive cycles of chemotherapy [33, 34]. A pilot study evaluating 77 non-diabetic patients who received at least three cycles of HEC or MEC suggested that DEX-induced diabetes occurred in approximately 20% of patients [35]. Only one of the studies included in the current meta-analysis reported tolerability data concerning DEX-related side effects [14], while most studies reported on any side effects that can be defined as associated with DEX. In light of this, the meta-analysis does not allow to draw any firm conclusions about the tolerability profile of the DEX-sparing strategy. Although additional studies are needed to show any improvement in patient’s safety resulting from the DEX-sparing strategy, its use can be reasonably expected to be cost saving when considering the overall cost of managing DEX-related side effects [7, 30–35].

## Conclusion

The current meta-analysis shows that DEX-sparing regimens do not result in any significant loss in anti-emetic protection against not only vomiting but also nausea during the delayed period in the settings of single-day MEC and AC HEC. The lack of efficacy data justifies the fact that the DEX-sparing strategy is not yet recommended for the control of CINV caused by cisplatin HEC [36]. Therefore, our data should lead clinicians to optimise use of DEX without compromising anti-emetic efficacy during the planned cycles of emetogenic chemotherapy. The DEX-sparing strategy can also be of utmost value in all patients who are at increased risk of corticosteroid-related side effects.

## Supplementary information

**Additional file 1: Table S1.** Studies rejected after screening.

**Additional file 2: Figure S1.** Risk of bias graph.

**Additional file 3: Figures S2-S6.** Forest plot of subgroup analyses.

## Data Availability

All data generated or analysed during this study are included in this published article and its supplementary information files.

## Abbreviations

5-HT_3_RA: 5-hydroxytryptamine type 3 receptor antagonist
AC: Anthracycline and cyclophosphamide
CINV: Chemotherapy-induced nausea and vomiting
CP: Complete protection
CR: Complete response
HEC: Highly emetogenic chemotherapy
MEC: Moderately emetogenic chemotherapy
NK-1RA: Neurochinin-1 receptor antagonist
NNH: Number needed to harm
OR: Odds ratio
RCT: Randomised controlled trial
RD: Risk difference
TC: Total control

## References

[1] Hilarius DL, Kloeg PH, van der Wall E, van den Heuvel JJ, Gundy CM, Aaronson NK (2012). Chemotherapy-induced nausea and vomiting in daily clinical practice: a community hospital-based study. Support Care Cancer.

[2] Schwartzberg L, Harrow B, Lal LS, Radtchenko J, Lyman GH (2015). Resource utilization for chemotherapy-induced nausea and vomiting events in patients with solid tumors treated with antiemetic regimens. Am Health Drug Benefits.

[3] Navari RM, Aapro M (2016). Antiemetic prophylaxis for chemotherapy-induced nausea and vomiting. N Engl J Med.

[4] Roila F, Molassiotis A, Herrstedt J, Aapro M, Gralla RJ, Bruera E (2016). 2016 MASCC and ESMO guideline update for the prevention of chemotherapy- and radiotherapy-induced nausea and vomiting and of nausea and vomiting in advanced cancer patients. Ann Oncol.

[5] Hesketh PJ, Kris MG, Basch E, Bohlke K, Barbour SW, Clark-Snow RA (2017). Antiemetics: American Society of Clinical Oncology clinical practice guideline update. J Clin Oncol.

[6] Grunberg SM (2007). Antiemetic activity of corticosteroids in patients receiving cancer chemotherapy : dosing, efficacy, and tolerability analysis. Ann Oncol.

[7] Vardy J, Chiew KS, Galica J, Pond GR, Tannock IF (2006). Side effects associated with the use of dexamethasone for prophylaxis of delayed emesis after moderately emetogenic chemotherapy. Br J Cancer.

[8] Rojas C, Slusher BS (2012). Pharmacological mechanisms of 5-HT_3_ and tachykinin NK_1_ receptor antagonism to prevent chemotherapy-induced nausea and vomiting. Eur J Pharmacol.

[9] Celio L, Niger M, Ricchini F, Agustoni F (2015). Palonosetron in the prevention of chemotherapy-induced nausea and vomiting: an evidence-based review of safety, efficacy, and place in therapy. Core Evid.

[10] Aapro M, Fabi A, Nolè F, Medici M, Steger G, Bachmann C (2010). Double-blind, randomised, controlled study of the efficacy and tolerability of palonosetron plus dexamethasone for 1 day with or without dexamethasone on days 2 and 3 in the prevention of nausea and vomiting induced by moderately emetogenic chemotherapy. Ann Oncol.

[11] Celio L, Frustaci S, Denaro A, Buonadonna A, Ardizzoia A, Piazza E (2011). Palonosetron in combination with 1-day versus 3-day dexamethasone for prevention of nausea and vomiting following moderately emetogenic chemotherapy: a randomized, multicenter, phase III trial. Support Care Cancer.

[12] Navari RM, Qin R, Ruddy KJ, Liu H, Powell SF, Bajaj M (2016). Olanzapine for the prevention of chemotherapy-induced nausea and vomiting. N Engl J Med.

[13] Roila F, Ruggeri B, Ballatori E, Del Favero A, Tonato M (2014). Aprepitant versus dexamethasone for preventing chemotherapy-induced delayed emesis in patients with breast cancer: a randomized double-blind study. J Clin Oncol.

[14] Ito Y, Tsuda T, Minatogawa H, Kano S, Sakamaki K, Ando M (2018). Placebo-controlled, double-blind phase III study comparing dexamethasone on day 1 with dexamethasone on days 2 and 3 with combined neurokinin-1 receptor antagonist and palonosetron in high-emetogenic chemotherapy. J Clin Oncol.

[15] Herrington JD, Jaskiewicz AD, Song J (2008). Randomized, placebo-controlled, pilot study evaluating aprepitant single dose plus palonosetron and dexamethasone for the prevention of acute and delayed chemotherapy-induced nausea and vomiting. Cancer.

[16] Likun Z, Xiang J, Yi B, Xin D, Tao ZL (2011). A systematic review and meta-analysis of intravenous palonosetron in the prevention of chemotherapy-induced nausea and vomiting in adults. Oncologist.

[17] Higgins JP, Altman DG, Gotzsche PC, Juni P, Moher D, Oxman AD (2011). The Cochrane Collaboration’s tool for assessing risk of bias in randomised trials. BMJ.

[18] Moher D, Liberati A, Tetzlaff J, Altman DG (2009). Preferred reporting items for systematic review and meta-analysis: the PRISMA statement. Ann Inter Med.

[19] Higgins JPT, Green S (editors). Cochrane Handbook for Systematic Reviews of Interventions Version 5.1 [updated March 2011]. The Cochrane Collaboration, http://www.handbook.cochrane.org. 2011. Accessed 26 Feb 2019.

[20] Sedgwick P (2013). Meta-analyses: heterogeneity and subgroup analysis. BMJ.

[21] Roscoe JA, Heckler CE, Morrow GR, Mohile SG, Dakhil SR, Wade JL (2012). Prevention of delayed nausea: a University of Rochester Cancer Center Community Clinical Oncology Program study of patients receiving chemotherapy. J Clin Oncol.

[22] Furukawa N, Kanayama S, Tanase Y, Ito F (2015). Palonosetron in combination with 1-day versus 3-day dexamethasone to prevent nausea and vomiting in patients receiving paclitaxel and carboplatin. Support Care Cancer.

[23] Matsuura M, Satohisa S, Teramoto M, Tanaka R, Iwasaki M, Nishikawa A (2015). Palonosetron in combination with 1-day versus 3-day dexamethasone for prevention of nausea and vomiting following paclitaxel and carboplatin in patients with gynecologic cancers: a randomized, multicenter, phase-II trial. J Obstet Gynaecol Res.

[24] Komatsu Y, Okita K, Yuki S, Furuhata T, Fukushima H, Masuko H (2015). Open-label, randomized, comparative, phase III study on effects of reducing steroid use in combination with palonosetron. Cancer Sci.

[25] Kosaka Y, Tanino H, Sengoku N, Minatani N, Kikuchi M, Nishimiya H (2016). Phase II randomized, controlled triaòl of 1 day versus 3 days of dexamethasone combined with palonosetron and aprepitant to prevent nausea and vomiting in Japanese breast cancer patients receiving anthracycline-based chemotherapy. Support Care Cancer.

[26] Bosnjak SM, Gralla RJ, Schwartzberg L (2017). Prevention of chemotherapy-induced nausea: the role of neurokinin-1 (NK_1_) receptor antagonists. Support Care Cancer.

[27] Okada Yuki, Oba Koji, Furukawa Naoto, Kosaka Yoshimasa, Okita Kenji, Yuki Satoshi, Komatsu Yoshito, Celio Luigi, Aapro Matti (2019). One‐Day Versus Three‐Day Dexamethasone in Combination with Palonosetron for the Prevention of Chemotherapy‐Induced Nausea and Vomiting: A Systematic Review and Individual Patient Data‐Based Meta‐Analysis. The Oncologist.

[28] Navari RM, Gray SE, Kerr AC (2011). Olanzapine versus aprepitant for the prevention of chemotherapy-induced nausea and vomiting: a randomized phase III trial. J Support Oncol.

[29] Waljee AK, Rogers MAM, Lin P, Singal AG, Stein JD, Marks RM (2017). Short term use of oral corticosteroids and related harms among adults in the United States: population based cohort study. BMJ.

[30] Zhao J, Dai YH, Xi QS, Yu SY (2013). A clinical study on insomnia in patients with cancer during chemotherapy containing high-dose glucocorticoids. Pharm.

[31] Rowbottom L, Stinson J, McDonald R, Emmenegger U, Cheng S, Lowe J (2015). Retrospective review of the incidence of monitoring blood glucose levels in patients receiving corticosteroids with systemic anticancer therapy. Ann Palliat Med.

[32] Eren OO, Ozturk MA, Oyan B (2014). Cancer-related fatigue: can it be due to adrenal suppression secondary to high-dose steroids used as antiemetic [letter]?. Support Care Cancer.

[33] Han HS, Park JC, Park SY, Lee KT, Bae SB, Kim HJ (2015). A prospective multicenter study evaluating secondary adrenal suppression after antiemetic dexamethasone therapy in cancer patients receiving chemotherapy: a Korean south west oncology group study. Oncologist.

[34] Nakamura M, Ishiguro A, Muranaka T, Fukushima H, Yuki S, Ono K (2017). A prospective observational study on effect of short-term periodic steroid premedication on bone metabolism in gastrointestinal cancer (ESPRESSO-01). Oncologist.

[35] Jeong Y, Han HS, Lee HD, Yang J, Jeong J, Choi MK (2016). A pilot study evaluating steroid-induced diabetes after antiemetic dexamethasone therapy in chemotherapy-treated cancer patients. Cancer Res Treat.

[36] Celio L, Bonizzoni E, Aapro M (2018). Is the dexamethasone-sparing strategy ready for cisplatin? Too early for an answer. J Clin Oncol.

