# Supplementary material for: Impact of dexamethasone-sparing regimens on delayed nausea caused by moderately or highly emetogenic chemotherapy: a meta-analysis of randomised evidence
Source: BMC Cancer. 2019 Dec 30;19:1268. doi: 10.1186/s12885-019-6454-y (PMC6937643; doi:10.1186/s12885-019-6454-y)
Supplement: Supplementary file 1 — Additional file 1: Table S1. Studies rejected after screening. [file 12885_2019_6454_MOESM1_ESM.docx]

**Table S1 Studies with the dexamethasone-sparing strategy rejected after screening**

| **No.** | **Reference** | **Reason for rejection** |
| --- | --- | --- |
| 1 | Celio L, et al. Palonosetron plus 1-day dexamethasone for the prevention of nausea and vomiting due to moderately emetogenic chemotherapy: effect of established risk factors on treatment outcome in a phase III trial. J Support Oncol 2012;10:65-71. | Publication with additional data not pertinent to the meta-analysis |
| 2 | Celio L, et al. Palonosetron plus single-dose dexamethasone for the prevention of nausea and vomiting in women receiving anthracycline/cyclophosphamide-containing chemotherapy: meta-analysis of individual patient data examining the effect of age on outcome in two phase III trials. Support Care Cancer 2013;21:565-73. | Publication with additional data not pertinent to the meta-analysis |
| 3 | Aapro M, et al. A randomized phase III study evaluating the efficacy and safety of NEPA, a fixed-dose combination of netupitant and palonosetron, for prevention of chemotherapy-induced nausea and vomiting following moderately emetogenic chemotherapy. Ann Oncol 2014;25:1328-33. | Inappropriate study design |
| 4 | Kitayama H, et al. Efficacy of palonosetron and 1-day dexamethasone in moderately emetogenic chemotherapy compared with fosaprepitant, granisetron, and dexamethasone: a prospective randomized crossover study. Int J Clin Oncol 2015;20:1051-56. | Inappropriate study design |
| 5 | Raftopoulos H et al. Comparison of an extended-release formulation of granisetron (APF530) versus palonosetron for the prevention of chemotherapy-induced nausea and vomiting associated with moderately or highly emetogenic chemotherapy: results of a prospective, randomized, double-blind, noninferiority phase 3 trial. Support Care Cancer 2015;23:723-32. | Inappropriate study design |
| 6 | Seol MY, et al. Transdermal granisetron versus palonosetron for prevention of chemotherapy-induced nausea and vomiting following moderately emetogenic chemotherapy: a multicenter, randomized, open-label, cross-over, active-controlled, and phase IV study. Support Care Cancer 2016;24:945-52. | Inappropriate study design |
| 7 | Celio L, et al. Should clinicians always administer dexamethasone beyond 24 h after chemotherapy to control delayed nausea and vomiting caused by moderately emetogenic regimens? Insight from the re-evaluation of two randomized studies. Support Care Cancer 2016;24:1025-34. | Publication with additional data not pertinent to the meta-analysis |
| 8 | Aapro L, et al. NEPA, a fixed oral combination of netupitant and palonosetron, improves control of chemotherapy-induced nausea and vomiting (CINV) over multiple cycles of chemotherapy: results of a randomized, double-blind, phase 3 trial versus oral palonosetron. Support Care Cancer 2017;25:1127-35. | Publication with additional data not pertinent to the meta-analysis |
