# Supplementary material for: Impact of dexamethasone-sparing regimens on delayed nausea caused by moderately or highly emetogenic chemotherapy: a meta-analysis of randomised evidence
Source: BMC Cancer. 2019 Dec 30;19:1268. doi: 10.1186/s12885-019-6454-y (PMC6937643; doi:10.1186/s12885-019-6454-y)

**Figure S1** Risk of bias graph: each risk of bias item presented as percentages across all included studies, comparing palonosetron and single-dose dexamethasone with palonosetron and 3-day dexamethasone.


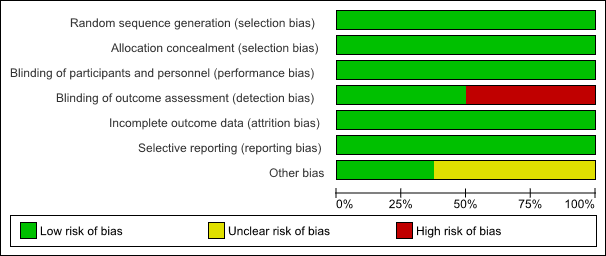

Supplement: Supplementary file 2 — Additional file 2: Figure S1. Risk of bias graph. [file 12885_2019_6454_MOESM2_ESM.docx]
