# Supplementary material for: Impact of dexamethasone-sparing regimens on delayed nausea caused by moderately or highly emetogenic chemotherapy: a meta-analysis of randomised evidence
Source: BMC Cancer. 2019 Dec 30;19:1268. doi: 10.1186/s12885-019-6454-y (PMC6937643; doi:10.1186/s12885-019-6454-y)

**Figure S2a** Forest plot of odds ratios for patients receiving palonosetron with 1-day or 3-day dexamethasone for prevention of chemotherapy induced nausea and vomiting, comparing MEC studies with AC studies: Complete response (CR) in the delayed period.

MEC, moderately emetogenic chemotherapy; AC, anthracycline and cyclophosphamide.


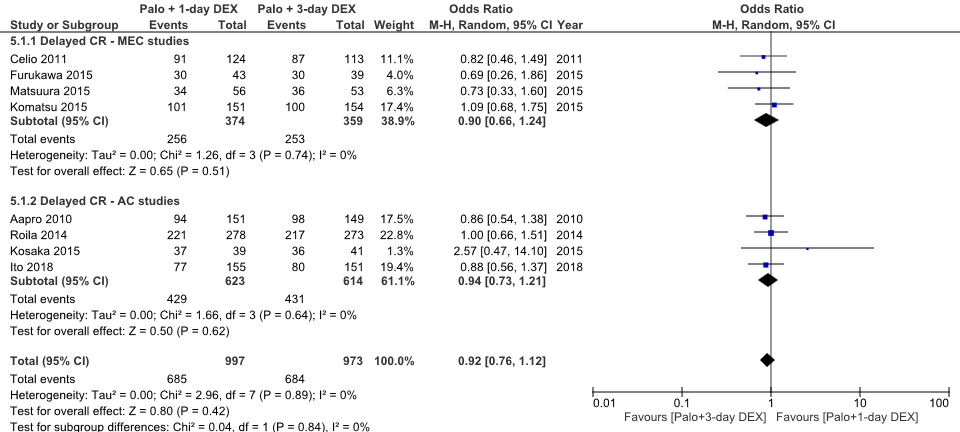


**Figure S2b** Forest plot of odds ratios for patients receiving palonosetron with 1-day or 3-day dexamethasone for prevention of chemotherapy induced nausea and vomiting, comparing MEC studies with AC studies: Complete protection (CP) in the delayed period.

MEC, moderately emetogenic chemotherapy; AC, anthracycline and cyclophosphamide.


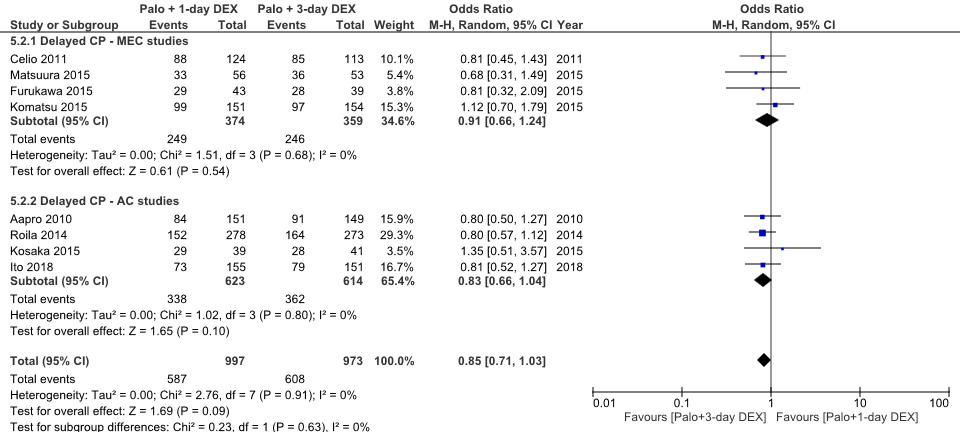


**Figure S2c** Forest plot of odds ratios for patients receiving palonosetron with 1-day or 3-day dexamethasone for prevention of chemotherapy induced nausea and vomiting, comparing MEC studies with AC studies: Total control (TC) in the delayed period.

MEC, moderately emetogenic chemotherapy; AC, anthracycline and cyclophosphamide.


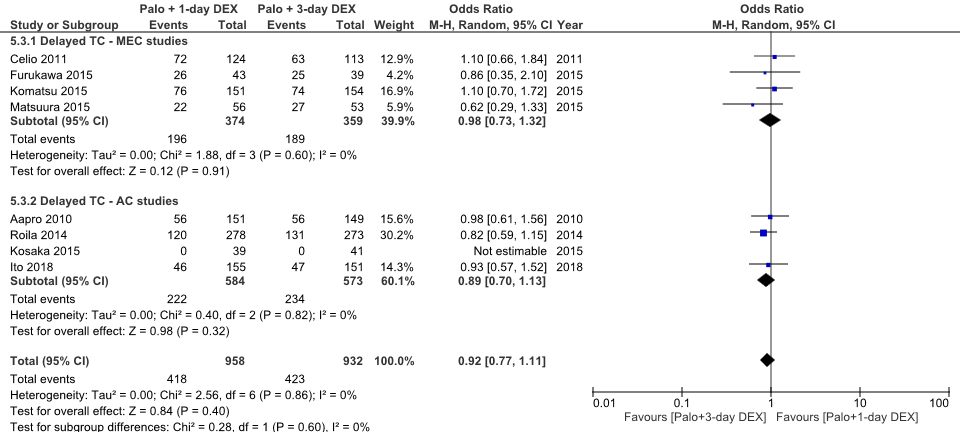


**Figure S3a** Forest plot of odds ratios for patients receiving palonosetron with 1-day or 3-day dexamethasone for prevention of chemotherapy induced nausea and vomiting, comparing unblinded studies with single-blind and double-blind studies: Complete response (CR) in the delayed period.


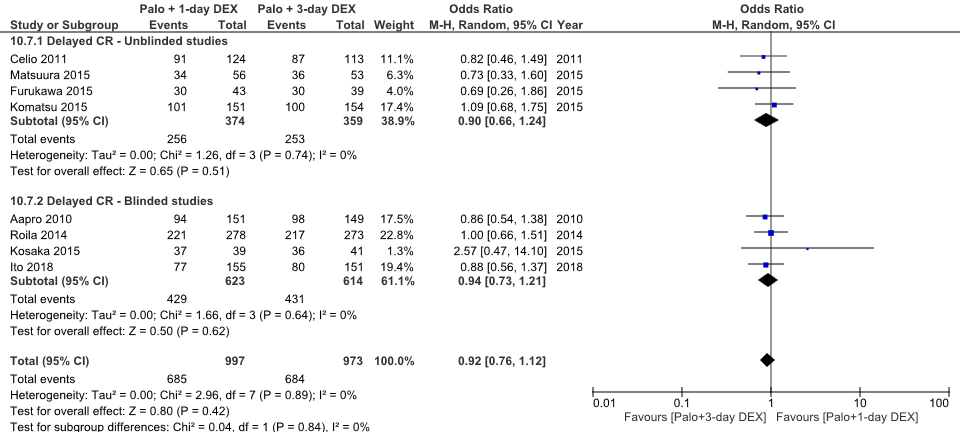


**Figure S3b** Forest plot of odds ratios for patients receiving palonosetron with 1-day or 3-day dexamethasone for prevention of chemotherapy induced nausea and vomiting, comparing unblinded studies with single-blind and double-blind studies: Complete protection (CP) in the delayed period.


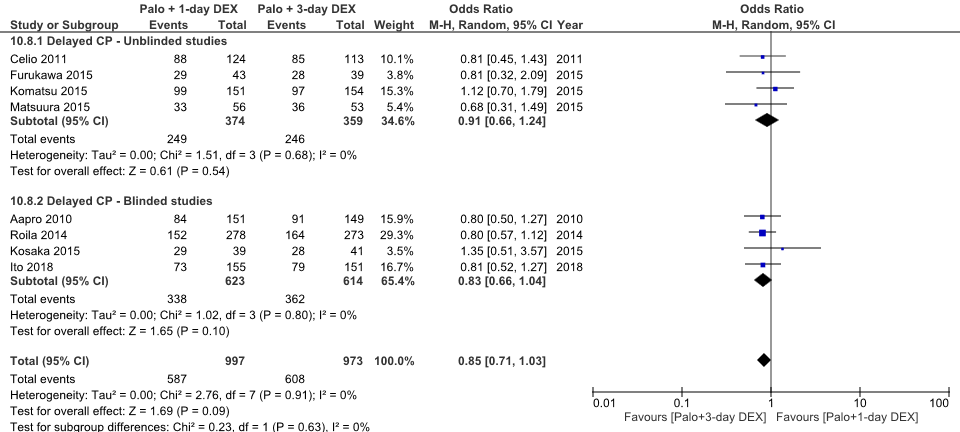


**Figure S3c** Forest plot of odds ratios for patients receiving palonosetron with 1-day or 3-day dexamethasone for prevention of chemotherapy induced nausea and vomiting, comparing unblinded studies with single-blind and double-blind studies: Total control (TC) in the delayed period.


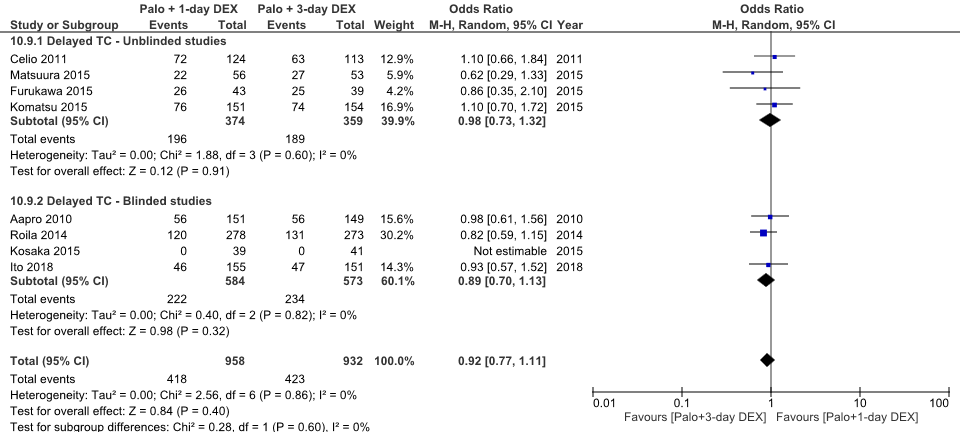


**Figure S4a** Forest plot of odds ratios for patients receiving palonosetron with 1-day or 3-day dexamethasone for prevention of chemotherapy induced nausea and vomiting, comparing small studies with large studies: Complete response (CR) in the delayed period.


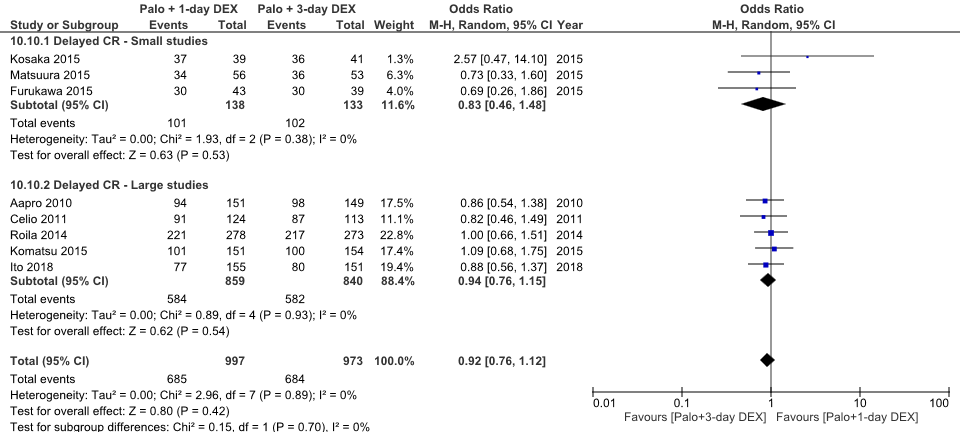


**Figure S4b** Forest plot of odds ratios for patients receiving palonosetron with 1-day or 3-day dexamethasone for prevention of chemotherapy induced nausea and vomiting, comparing small studies with large studies: Complete protection (CP) in the delayed period.


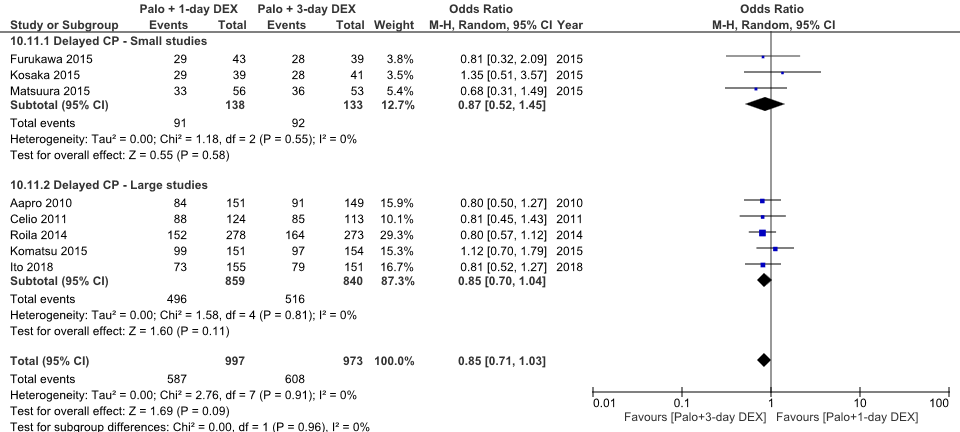


**Figure S4c** Forest plot of odds ratios for patients receiving palonosetron with 1-day or 3-day dexamethasone for prevention of chemotherapy induced nausea and vomiting, comparing small studies with large studies: Total control (TC) in the delayed period.


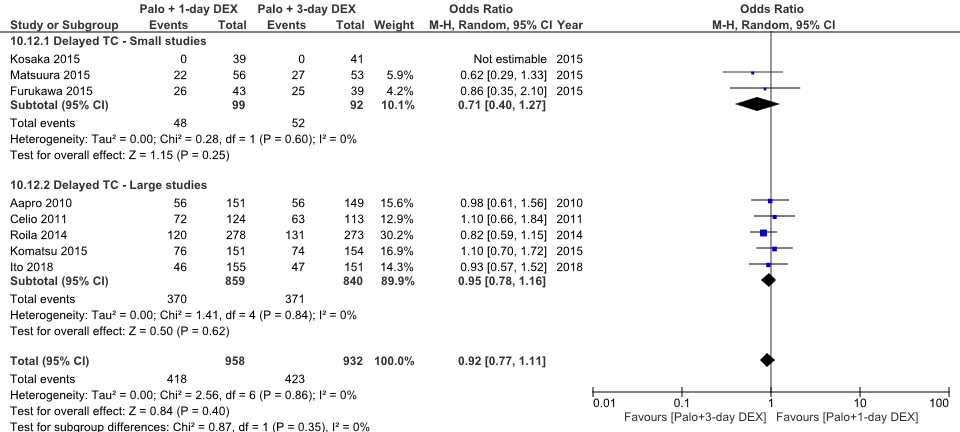


**Figure S5a** Forest plot of odds ratios for patients receiving palonosetron with 1-day or 3-day dexamethasone for prevention of chemotherapy induced nausea and vomiting, comparing mixed studies with only-female studies: Complete response (CR) in the delayed period.


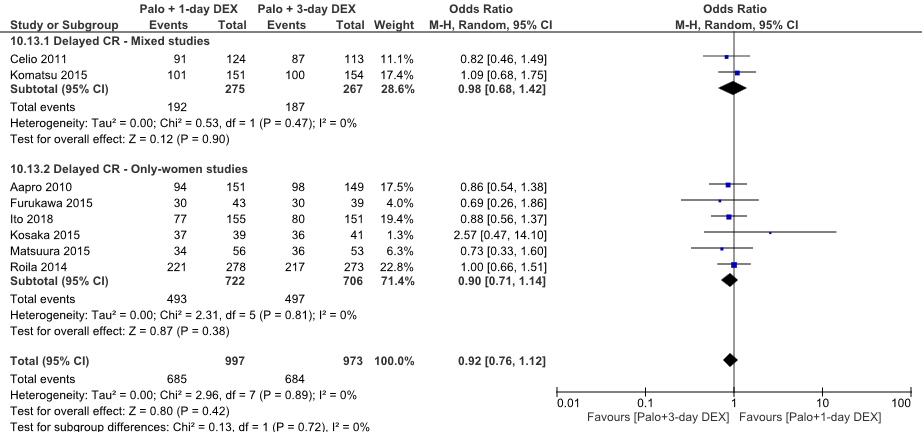


**Figure S5b** Forest plot of odds ratios for patients receiving palonosetron with 1-day or 3-day dexamethasone for prevention of chemotherapy induced nausea and vomiting, comparing mixed studies with only-female studies: Complete protection (CP) in the delayed period.


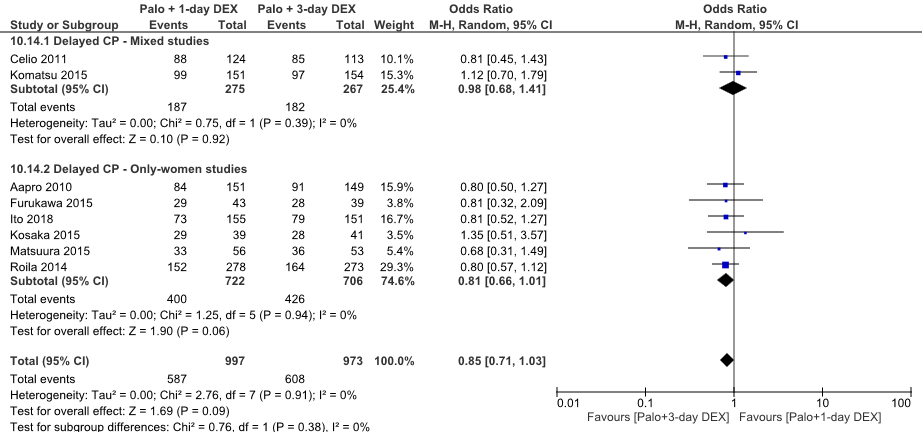


**Figure S5c** Forest plot of odds ratios for patients receiving palonosetron with 1-day or 3-day dexamethasone for prevention of chemotherapy induced nausea and vomiting, comparing mixed studies with only-female studies: Total control (TC) in the delayed period.


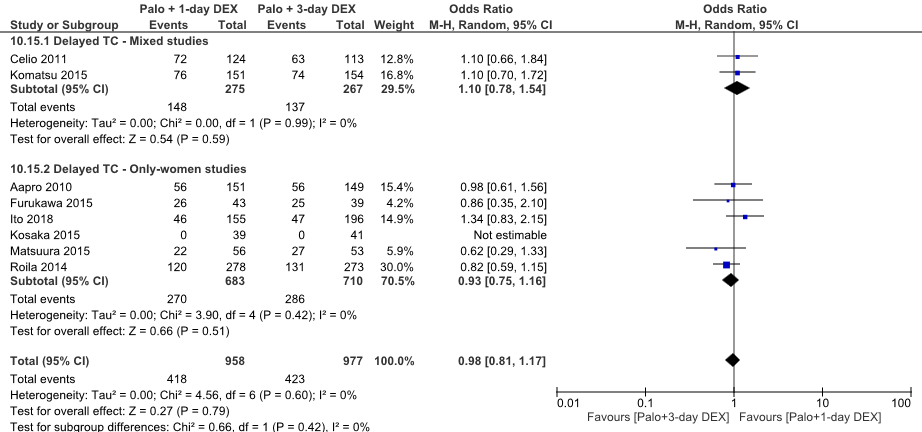


**Figure S6a** Forest plot of odds ratios for patients receiving palonosetron with 1-day or 3-day dexamethasone for prevention of chemotherapy induced nausea and vomiting, comparing studies without NK-1RA with studies including NK-1RA: Complete response (CR) in the delayed period.

NK-1RA, neurokinin-1 receptor antagonist.


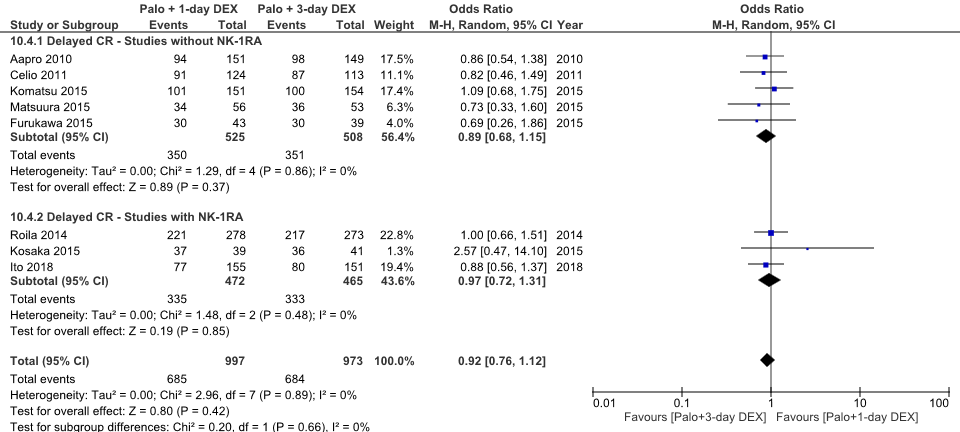


**Figure S6b** Forest plot of odds ratios for patients receiving palonosetron with 1-day or 3-day dexamethasone for prevention of chemotherapy induced nausea and vomiting, comparing studies without NK-1RA with studies including NK-1RA: Complete protection (CP) in the delayed period.

NK-1RA, neurokinin-1 receptor antagonist.


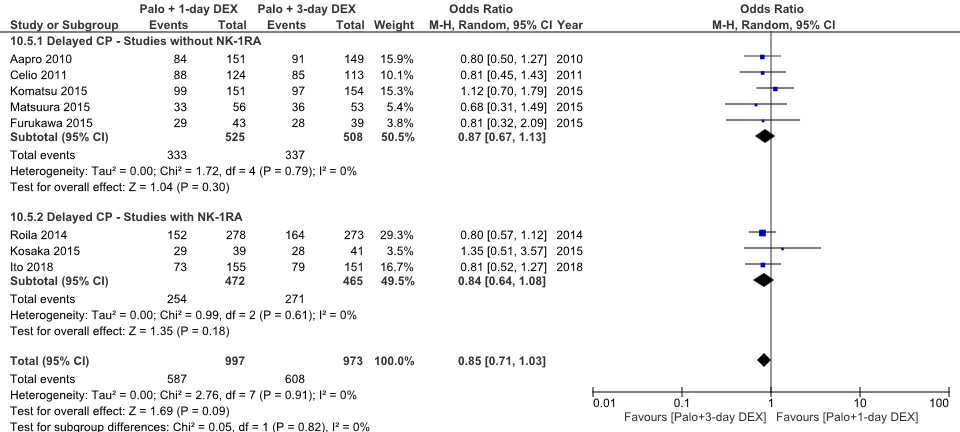


**Figure S6c** Forest plot of odds ratios for patients receiving palonosetron with 1-day or 3-day dexamethasone for prevention of chemotherapy induced nausea and vomiting, comparing studies without NK-1RA with studies including NK-1RA: Total control (TC) in the delayed period.

NK-1RA, neurokinin-1 receptor antagonist.


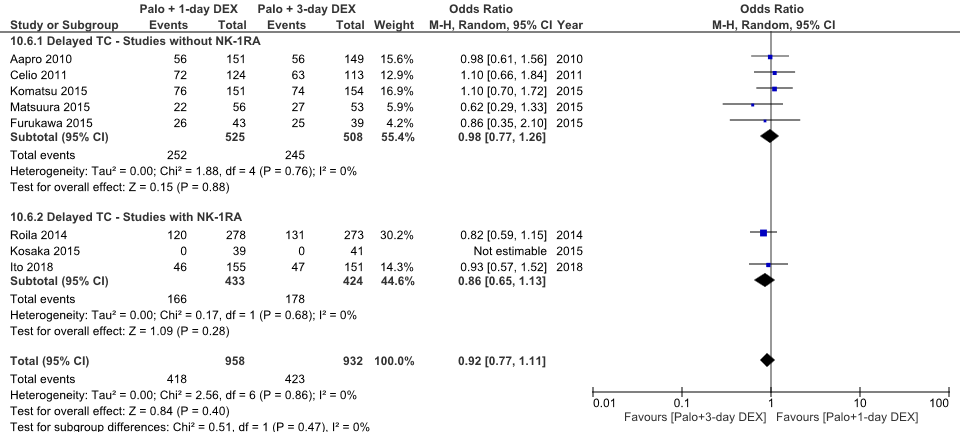

Supplement: Supplementary file 3 — Additional file 3: Figures S2-S6. Forest plot of subgroup analyses. [file 12885_2019_6454_MOESM3_ESM.docx]
